# Beneficial Effects of Isoproterenol and Quinidine in the Treatment of Ventricular Fibrillation in Brugada Syndrome

**DOI:** 10.1155/2015/753537

**Published:** 2015-06-11

**Authors:** Melissa Dakkak, Khyati Baxi, Ambar Patel

**Affiliations:** Departments of Cardiovascular Diseases and Internal Medicine, University of Florida Health, Jacksonville, FL 32209, USA

## Abstract

The use of an implantable cardiac defibrillator has been advocated as the only effective treatment for the management of ventricular fibrillation (VF) in patients with Brugada Syndrome (BrS). However, this device is only useful for terminating VF. Intermittent and/or recalcitrant VF for which lifesaving cardioversion occurs is a problematic situation in this patient population. The immediate use of appropriate antiarrhythmics in the acute setting has proven to be lifesaving. Quinidine has been well established as an effective antiarrhythmic in BrS, while isoproterenol (ISP) has had some recognition as well. The addition of drug therapy to prevent the induction of these arrhythmias has been shown to reduce the morbidity and mortality associated with BrS. It was proven to be especially effective in the presence of early repolarization, evidenced by the reduction or normalization of the early repolarization pattern on ECG. Thus, for the prophylactic management and long term suppression of VF in BrS, further prospective studies should be performed to determine the effectiveness of quinidine and ISP in this patient population.

## 1. Introduction

The use of an implantable cardiac defibrillator in patients with Brugada Syndrome (BrS) has been advocated as the first line therapy for managing recalcitrant ventricular fibrillation (VF). However, this device is only useful for terminating VF. Intermittent and/or refractory VF for which lifesaving cardioversion occurs is a problematic situation in this patient population. After an initial adverse arrhythmic event, such as cardiac arrest or syncope due to VF, approximately 60% of symptomatic patients will have a recurrent arrhythmic event within the next 4 years [1, 2]. Therefore, the addition of antiarrhythmic suppression therapy may reduce the morbidity and mortality associated with BrS. At this time, no pharmacologic intervention has been proven to be effective in reducing the risk of initial cardiac arrest from VF [3]. This case and literature review will focus on the effectiveness of isoproterenol (ISP) and quinidine in preventing VF in patients with BrS.

## 2. Case

**Figure 1:**
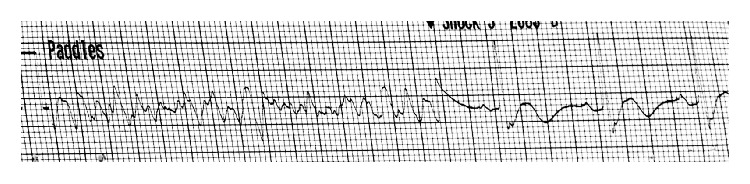
Electrocardiography strip demonstrating ventricular fibrillation with restoration of normal sinus rhythm after one cardioversion shock.

**Figure 2:**
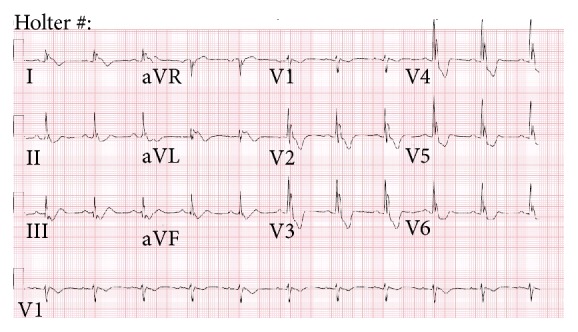
Baseline ECG on admission demonstrates interventricular conduction delay, QRS notching, and ST-T wave abnormalities.

**Figure 3:**
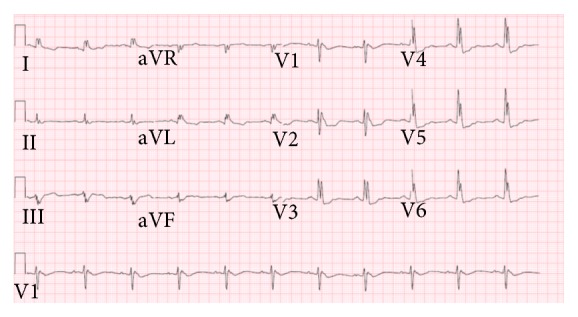
Baseline Electrocardiography during sinus rhythm showing coved-type ST segment in leads V1 and V2.

**Figure 4:**
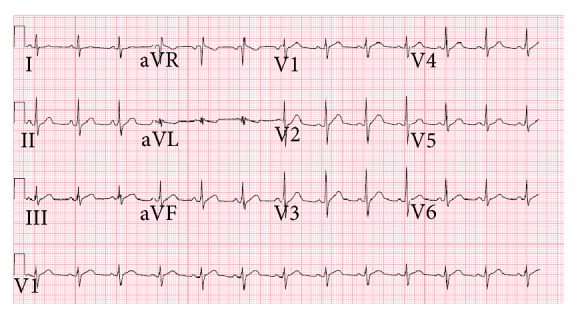
Electrocardiogram after Isoproterenol infusion and oral quinidine gluconate administration showing resolution of QRS notching and no J point elevation.

A 25-year-old African American male with no significant past medical or family history of sudden cardiac arrest (SCA) presented with VF. Electrocardiography (ECG) strip on arrival demonstrated VF (Figure 1). In the emergency department, the patient continued to have VF refractory to several antiarrhythmic agents, including amiodarone, esmolol, and lidocaine used alone or in combination. Physical examination while the patient was temporarily in sinus rhythm showed an obese afebrile male with no chest wall trauma and normal cardiac and pulmonary examination. Initial ECG demonstrated interventricular conduction delay with nonspecific ST-T wave changes as well as early repolarization pattern (ER) with QRS notching (Figure 2). Laboratory abnormalities included low magnesium (1.6 mg/dL) and potassium (3.3 mmol/L) levels, which were adequately replaced. The transthoracic echocardiogram showed a preserved left ventricular ejection fraction (EF) of 60–65% with normal wall motion, normal valvular function, and otherwise normal structure. The patient continued to have innumerable episodes of VF requiring a total of 63 shocks. Upon infusion of the beta-agonist ISP, an ECG performed during a temporary sinus rhythm showed a right bundle branch block morphology with a coved ST segment and negative T wave deflections in leads V1 and V2 (Figure 3). Urine drug screen was negative. The patient was continued on ISP at rate of 0.5 mcg/min. Once the patient status stabilized, ISP was weaned off and he was transitioned to oral quinidine gluconate 324 mg every 8 hours. No further arrhythmias occurred. The ECG on discharge showed near normalization of the ER pattern with resolution of the QRS notching and no J point elevation (Figure 4). The patient underwent further comprehensive testing including a coronary angiography, which demonstrated normal coronary arteries. Magnetic Resonance Imaging with and without gadolinium enhancement showed no myocardial scarring, inflammation, or infiltration, with a preserved EF of 62%. A diagnosis of Brugada syndrome (BrS) was established based on the characteristic ECG pattern in conjunction with documented VF. Prior to discharge, the patient received an implantable cardioverter defibrillator (ICD) for secondary prevention in conjunction with oral quinidine 324 mg every 8 hours for VF suppression. He has been free of arrhythmias for 7 months.

## 3. Discussion

BrS is diagnosed by characteristic ECG pattern in conjunction with at least one of the following: documented VF, self-terminating polymorphic VT, family history of sudden cardiac death, coved-type ECG pattern in family members, VF inducibility during electrophysiological study, syncope, or nocturnal agonal respirations [4, 5]. 

*Criteria for Brugada Syndrome*

ECG criteria

Type 1: elevation of the J point and coved-type ST segment elevation >2 mm followed by an inverted T wave that occurs spontaneously,
Type 2: saddle back-type ST segment elevation of >2 mm followed by either a positive T wave or biphasic T wave,
Type 3: either saddle back-type or coved-type ST segment elevation >1 mm.

Plus at least one of the following

 (i) documented VF,
 (ii) self-terminating polymorphic VT,
 (iii) family history of sudden cardiac death,
 (iv) coved-type ECG pattern in family members,
 (v) VF inducibility during EP study,
 (vi) syncope,
 (vii) nocturnal agonal respirations.

**Figure 5:**
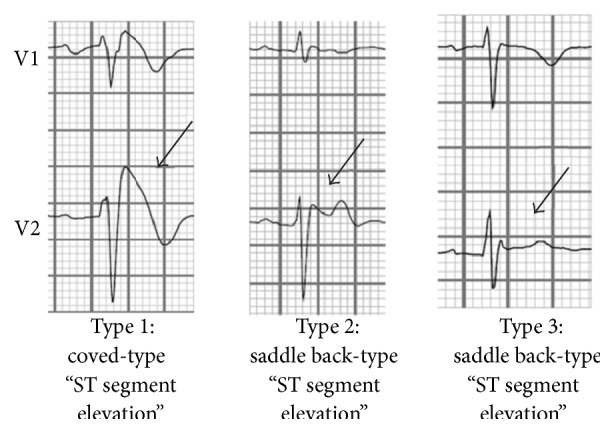
Three types of ECG pattern associated with BrS.

There are 3 types of BrS based on ECG characteristics. Type 1 ECG pattern shows pronounced elevation of the J point and coved-type ST segment elevation ≥2 mm followed by an inverted T wave that occurs spontaneously. Type 2 ECG pattern consists of saddle back-type ST segment elevation of ≥2 mm followed by either a positive T wave or biphasic T wave. Lastly, type 3 ECG pattern shows either saddle back-type or coved-type ST segment elevation ≥1 mm (Figure 5). In contrary to type 1, type 2 and type 3 may not occur spontaneously and thus require a pharmacological challenge test [4, 6].

The prevalence of BrS appears to be low in the general population and occurs predominantly in young male adults less than 40 years of age. No precise data are available on the epidemiology of BrS. However, its prevalence is much higher in Asian and South Asian countries, reaching 0.5–1 per 1000. BrS is 8–10 times more prevalent in men than in women. Approximately 20% of BrS patients have been shown to have a mutation located in the SCN5A gene, which produces a complete loss of function of the cardiac sodium channel [4, 7].

Risk stratification is paramount in BrS since no medications have been proven to be effective in increasing the risk of ventricular arrhythmias. Patients who have experienced an episode of SCA are at the highest risk of arrhythmia recurrence. The presence of a spontaneous type I ECG associated with history of syncope defines the second highest risk group. Conversely, asymptomatic patients with spontaneous ECG characteristics, such as those with type 2 and 3 Brugada pattern, can be considered for an electrophysiology study for further risk stratification [8, 9]. Guidelines for genetic testing in patients with suspected BrS are not definitive [10]. As it stands, ICD implantation is advocated to be the first line therapy in SCA in BrS survivors [11].

Additionally, the combination of fractionated-QRS (f-QRS) with early repolarization (ER) abnormalities is useful for identifying high- and low-risk patients [12]. ER pattern on ECG is indicated by the presence of an elevation of the QRS-ST junction (J point) in at least two leads. The amplitude of J point elevation has to be at least 1 mm (0.1 mV) above the baseline level, either as QRS slurring depicted as a smooth transition from the QRS segment to the ST segment or notching represented by a positive J deflection inscribed on the S wave in the inferior lead (II, III, and aVF), lateral lead (I, aVL, and V4 to V6), or both [13]. An ER pattern is currently considered to be a benign electrocardiographic phenomenon affecting 2% to 5% of the general population and is most commonly observed in young men. However, recently, an ER pattern has been shown to be an additional risk marker for VF development, especially in inferolateral leads, in patients with BrS [14, 15]. The study performed by Tokioka et al. showed that the combination of f-QRS and inferolateral ER pattern was associated with the development of VF in these patients. Furthermore, repolarization abnormalities were independently associated with VF development. Moreover, VF and SCA episodes during follow-up and a history of VF episodes were more frequently observed in patients with an ER pattern than in those without an ER pattern (*p* = 0.001 and *p* = 0.005, resp.) [12].

The pathophysiology of BrS is complex but does impact the selection of antiarrhythmic medications to use for its management. The defective myocardial sodium channels reduce sodium inflow currents and consequentially reduce the duration of the normal action potential. Phase 0 of the action potential, which correlates with influx of sodium, is blunted and this results in reduction of calcium inflow and shortening of phase 2. The cells may therefore fail to conduct the action potential due to a shortened refractory period, which may give rise to localized reentry circuits and the potential of arrhythmias in the presence of ventricular premature beats. The effect is more pronounced when there are normal and abnormal sodium channels in the same tissue with heterogeneity of refractory periods [16]. Attacks of VF usually occur at the night during sleep during which there is an increase in vagal stimulation causing an increase in outward current and decrease in inward current, leading to a shortening of the action potential duration and excitation. Beta-adrenergic stimulation through the sympathetic nervous system induces the contrary, by increasing inward calcium current and attenuating the excess outward current and thus counterbalancing the changes in membrane potential [17].

Despite their anecdotal success and proven efficacy, ISP and Quinidine are Class IIa recommendations according to the HRS/EHRA/APHRS and Class IIa and Class IIb recommendations according to ACC/AHA, respectively, for the management of arrhythmic storms such as VF storm [18, 19].

Quinidine, a class IA antiarrhythmic, has been established as an effective drug in the management of BrS. It exerts its beneficial effects in BrS by inhibiting the outward current, thereby restoring electrical homogeneity. In addition, it prolongs ventricular refractoriness [20]. The study by Belhassen et al. demonstrated that quinidine had an 88% success rate in preventing VF induction in BrS patients with inducible VF. Furthermore, quinidine was effective in preventing spontaneous VF during follow-up ranging from 6 months to 18 years [20]. In a case series performed by Márquez et al., quinidine was effective in suppressing ventricular arrhythmias during a mean follow-up time of 4 years. Long-term use of quinidine was well tolerated at a low dose of <600 mg/d while maintaining an effectiveness of 85% [21]. The limiting factor for long-term compliance included noncardiac side effects such as abdominal cramping, diarrhea, cinchonism and anticholinergic effects and proarrhythmia in the setting of electrolyte abnormalities [22].

In contrast, ISP has not been as well studied as quinidine, though its efficacy has been documented. ISP is a beta-adrenergic agonist that increases intracellular calcium in order to stabilize and restore the dome in phase 2 of the action potential and reduce the electrical heterogeneity responsible for BrS. This stability reduces the susceptibility to VF triggered by premature beats. In addition to successfully terminating and suppressing the refractory ventricular fibrillation as in the above case, it has also been shown to normalize the electrocardiographic pattern and prevent ventricular fibrillation induction during electrophysiological study. ISP was effective in suppressing VF in a 36-year-old male with BrS and was also associated with the disappearance of the short-coupled premature ventricular beats, which trigger VF [23]. Its effect is also confirmed in a case series by Watanabe et al., in which ventricular arrhythmias were successfully abolished after the infusion of ISP in six patients with BrS. The suppressive effect continued for three days after the termination of the infusion. However, one patient had recurrent ventricular arrhythmias following the end of the isoproterenol infusion. The addition of quinidine was effective in terminating the arrhythmias. Thus, the direct effect of ISP on the myocardium to increase inward current is important for therapeutic effects in patients with BrS [17].

The infusion of ISP followed by the oral administration of quinidine used for the management of VF in BrS has been documented in very few case studies and small studies. A recent case report by Furniss confirmed its efficacy in terminating and preventing VF in a 3-year-old male with BrS who has been event-free for 1 year [16]. This was preceded by a case by Jongman et al, in which a 45-year-old male with BrS type 2 presented with numerous ICD shocks for VF [22]. A study performed in 2007 by Ohgo et al. demonstrated that ISP infusion was successful in terminating VF storm in the acute setting in patients with BrS. These patients were then successfully transitioned to oral antiarrhythmics including quinidine for chronic suppression of VT/VF [24]. Therefore, a prospective study to determine the long-term efficacy of ISP infusion followed by the administration of oral quinidine in remaining arrhythmia-free in BrS patients with an ICD would be of interest.

A multicenter study performed by Haïssaguerre et al. demonstrated the efficacy of ISP and quinidine to abolish and prevent recurrences of VF associated with early repolarization abnormality in the inferolateral leads. ISP was infused in 7 patients during repetitive episodes of VF at a rate of 1 to 5 *μ*g/min, which eliminated all arrhythmias when the sinus heart rate was increased to above 120 beats/min. Any attempt to reduce the infusion and heart rate was associated with recurrence of VF in 3 of the patients. Isoproterenol was infused for a period ranging from 6 h to 5 days. In addition, quinidine (in 3) or hydroquinidine (in 6) was totally successful in 9 of 9 patients in decreasing the number of recurrent VF from a mean of 33 episodes to nil with follow-up of 25 ± 18 months on therapy. The study concluded that ISP and quinidine both reduced the ER pattern or restored a normal ECG, which also occurred in our patient. This demonstrates that ISP and quinidine are effective in preventing the recurrence of VF associated with ER abnormality. Thus, this study confirmed that the infusion of ISP can successfully manage electrical storms as a lifesaving therapy, while the oral administration of quinidine is effective chronically on a long-term basis [12].

## 4. Conclusion

In an otherwise young healthy male with no significant cardiac risk factors, hereditary channelopathies such as BrS should be higher in the differential. The immediate use of appropriate antiarrhythmics has proven to be lifesaving. Quinidine has been well established as an effective antiarrhythmic in BrS, while ISP has had some recognition as well. Interestingly, the use of isoproterenol and quinidine has been successful in long-term prevention of VF in case reports and studies. It has been proven to be especially effective in the presence of ER pattern evident by the reduction or normalization of this pattern on ECG. Thus, further prospective studies should be performed to determine the effectiveness of quinidine and ISP therapy in early management and long-term suppression of VF in the BrS population.
